# The effectiveness of internal cohesion psychotherapy in treating young clients with depression and anxiety disorders: The role of developmental assets in Kosovo context

**DOI:** 10.3389/fpsyg.2022.1005709

**Published:** 2022-10-12

**Authors:** Fitim Uka, Shkumbin Gashi, Arlinda Gashi, Diellza Gllogu, Arian Musliu, Albina Krasniqi, Albina Statovci, Vanesa Sopjani, Veronë Perçuku, Irma Sadikovic, Nora Wiium

**Affiliations:** ^1^Department of Psychology, University of Prishtina, Prishtina, Kosovo; ^2^Research Department, Empatia Multidisciplinary Clinic, Prishtina, Kosovo; ^3^Munich Center of the Learning Sciences, Ludwig Maximilians University, Munich, Bavaria, Germany; ^4^Departement Gesundheit, Berner Fachhochschule, Bern, Switzerland; ^5^Faculty of Education, University of Prishtina, Prishtina, Kosovo; ^6^Psycho-Social and Medical Research Center, Prishtina, Kosovo; ^7^Faculty of Psychology, University of Bergen, Bergen, Norway

**Keywords:** internal cohesion psychotherapy, developmental assets, youth mental health, Kosovo, psychotherapy

## Abstract

The positive youth development approach (PYD) is widely used as a meaningful framework to guide research, policy, and intervention, to support young people to develop their full potential. Psychotherapy, on the other hand, is a verbal and psychological procedure that can be a suitable solution to mental health concerns, which are prevalent among youth. This study aimed to explore the potential role of developmental assets in treating clients with depression and anxiety disorders using Internal Cohesion Psychotherapy (ICP). In total, 10 young people who took at least five sessions of ICP were part of this study. In-depth semi-structured interviews were conducted to gather information about clients’ experiences with ICP and their perception/opinions on the presence of developmental assets in their lives. The results confirm the effectiveness of ICP in treating depression and anxiety, while clients acknowledge the role of developmental assets in their psychotherapeutic process. The current study has important theoretical, practical, and research implications. It provides evidence on how clients use their developmental assets in maximizing the effectiveness of the ICP process. The usage of developmental assets to enhance the effectiveness of psychotherapy opens a new path for further research and can serve as a foundation ground for intervention on mental health among youth.

## Introduction

Mental health problems among young people are a growing public health concern. Several studies in the last 20 years shows a high prevalence of depression and anxiety among adolescents and young adults, ranging between 4–32% and 3.7–14%, respectively (Ayuso-Mateos et al., 2001; Costello et al., 2005; Jane Costello et al., 2006; Balazs et al., 2012; Economou et al., 2013; Ormel et al., 2015; Gustavson et al., 2018; Moreira de Sousa et al., 2018). A meta-analytic study confirmed that 14.3% of young adults experience depression and 19.1% experience anxiety (Barker et al., 2019). Research conducted in Kosovo context indicate that the prevalence of anxiety among youth ranges from 33.6% (Kamberi et al., 2019), whereas another study (Arenliu et al., 2016) shows that the prevalence of depressive symptoms among Kosovo population is higher than western European countries. Although the determinants of emotional disorders vary, age, unemployment, low economic status, marital status, and educational status were found to correlate with depression and anxiety (Steptoe et al., 2007; Mylona et al., 2014; Asselmann and Beesdo-Baum, 2015; Stathopoulou et al., 2018). On the other hand, there are many consequences of anxiety and depression, including social exclusion, discrimination, and stigma (affecting readiness to seek help), which can result in educational difficulties, risk-taking behaviors, physical ill-health, and human rights violations (World Health Organization, 2022). Altogether, the prevalence of depression and anxiety as well as their consequences among the youth population, show the need for increased psychological assessment, monitoring, and intervention.

A new line of intervention aiming to address the issue of emotional disorders is based on the positive youth development approach (PYD). PYD is widely applied as a meaningful framework to guide research, policy, and intervention, to support and enhance the opportunities for young people to develop their full potential (Cabrera and Leyendecker, 2017; Petersen et al., 2017; Dimitrova et al., 2018, 2021; Fernandes et al., 2021; Wiium et al., 2021). Developmental assets represent the most influential model of PYD (Roehlkepartain and Blyth, 2019), where both positive features of developmental ecologies with personal skills, competencies, and values are linked.

There are 40 developmental assets identified, consisting of 20 internal assets and 20 external assets, which represent values, relationships, resources, and skills (Benson et al., 2011). Internal assets are considered as an individual’s skills and competencies, which include a commitment to learning (motivation to succeed, learning commitment, homework, connection to the school, reading for pleasure), positive values (care, equality and social justice, integrity, honesty, responsibility, self-control), social competencies (planning and decision-making, interpersonal competencies, cultural competencies, appropriate conflict resolution), and positive identity (self-esteem, positive opinion of personal future). External assets are considered as all the resources of the environment, such as support (family support, positive family relationships, other relationships with adults, a caring neighborhood, a favorable school climate, parental involvement in schooling), boundaries, and expectations (those within the family, those within school, those within neighborhoods, adult role models, positive peer influence, high expectations), empowerment (community values, helping others, security), and constructive use of time (creative activities, youth programs, religious community, time spent at home; Benson et al., 2011).

Evidence-based intervention programs have been inspired by the positive results of developmental assets, which are negatively related to risky behaviors, sadness, and suicide attempts and can serve as protective factors for substance use disorders and mental health (Dimitrova et al., 2018; Adams et al., 2019; Substance Abuse and Mental Health Services Administration, 2021; Uka et al., 2021; Wiium et al., 2021). Furthermore, it is shown that the external assets are negatively correlated with depression, anxiety, and social dysfunction (Ismail et al., 2015), whereas the internal developmental assets are shown to be a better predictor of social, emotional, and psychological well-being than the external assets (Manrique-Millones, 2021). Findings from Kosovo context show that caring, empathy, positive identity and self-esteem are prevalent among young people (Fernandes et al., 2021), which are linked to positive development. In general, existing evidence indicates that the more developmental assets, the less the risk for mental health disorders.

Beyond the effects of developmental assets, the emotional experiences of individuals can act as potential contributors to a variety of psychological disorders. Meanwhile, psychotherapy is a verbal and psychological procedure that can be considered a suitable solution for treating mental health problems. Different types of psychotherapy are designed to provide symptom relief, reduce symptomatic episodes, enhance the quality of life, promote adaptive functioning in work/school and relationships, increase the likelihood of making healthy life choices, and offer other benefits established by the collaboration between client/patient and psychologist (Burlingame, et al., 2003; APA Presidential Task Force on Evidence-Based Practice, 2006; Kösters, et al., 2006; American Group Psychotherapy Association, 2007; Wampold, 2007, 2010, Carr, 2009a,b; Shedler, 2010; Tasca et al., 2015; Cook et al., 2017). At least 500 different types of psychotherapy exist (Norcross, 2005) and almost all types of psychotherapy tend to address depression and anxiety among young adults. In recent years, one of the prominent approaches in integrative psychotherapies is a more inclusive attitude toward the different psychotherapeutic models. There is an increasing number of psychotherapists, who identify themselves with an integrative approach to psychotherapy (Boswell et al., 2010; Stricker and Gold 2011; Zarbo et al., 2016).

Among many existing psychotherapies, Internal Cohesion Psychotherapy (ICP) is a recently developed integrative psychotherapy approach. ICP integrates the best theory and practices from different perspectives and the overarching goal is to reach internal cohesion (Uka, 2022; Uka, F. (n.d.)). The core components of Internal Cohesion Psychotherapy (ICP) are systems (intrapersonal, interpersonal, professional, and spiritual), which represent important relations between individuals and time. Internal cohesion is considered the psychological state, in which clients tend to have a better relationship with all the systems and among three times (the past, the present, and the future). Therefore, internal cohesion is considered a condition in which (1) the person can communicate openly with each system and accept the past, address and find solutions for the open conflicts related to each system in the past, and (2) establish healthy relations with each of the four systems in the present and (3) establish real and achievable expectations for the future relations with each system (Uka, 2022).

Theoretically, developmental assets are related to Internal Cohesion Psychotherapy in many ways. The intrapersonal system of ICP, which is considered as the communication of an individual with oneself and has core components such as self-regulation, self-esteem, and motivation, is similar to internal assets, which include an individual’s skills and competencies. Alternatively, the external assets, especially the support perceived or received from significant others, are related to the interpersonal system of ICP. Both healthy and unhealthy mental, emotional, and behavioral development are influenced by young adults’ intrapersonal and interpersonal relations (internal and external assets). These influences include physical, social, and other experiences, an individual’s sleep, nutrition, and physical activity, peer behavior, parental behavior, and societal characteristics (e.g., poverty, law- and policy-driven factors, systemic racism, and discrimination). However, less is known about the role of internal and external assets in psychotherapy. In this study, we aimed to explore the potential role of developmental assets in treating clients with depression and anxiety disorders using Internal Cohesion Psychotherapy. We have employed a qualitative approach to identify the role of both internal and external assets on the effectiveness of Internal Cohesion Psychotherapy in treating clients with anxiety and depression among young adults living in Kosovo.

## Materials and methods

### Participants

The current study included 10 participants (*N* = 10; *M*_age_ = 26.10, *SD* = 5.28), 9 female and 1 male, who showed symptoms of depression and anxiety disorders and were treated with Internal Cohesion Psychotherapy (ICT). In total, 7 of the participants held a bachelor’s degree, 2 had a master’s degree whereas one participant had a high school level education. Participants were from different regions of Kosovo and took psychotherapy services in the “Empatia” clinic in Prishtina. The eligibility criteria included young adults who showed symptoms of depression and anxiety and were treated using ICP. The eligible participants of this study were clients who took more than five sessions of psychotherapy and completed the therapeutic process. The average session frequency was once a week with a duration of 45–60 min. All participants were native Albanian speakers from the Republic of Kosovo.

### Instruments

The data were collected using two interviewing protocols. The first protocol “effectivity of Internal Cohesion Psychotherapy” included questions related to the participant’s perception of their psychotherapy experience, the overall evaluation of the effectiveness of psychotherapy, and the role of the psychotherapist in addressing issues related to four systems of ICP, such as intrapersonal, interpersonal, professional and spiritual along three time-perspectives (the past, the present and the future). The PYD protocol (Search Institute, 2004) was adopted in the Albanian language using the translation-back translation procedure. The protocol included questions related to the presence of external (support, empowerment, boundaries & expectations, constructive use of time) and internal assets (commitment to learning, positive values, social competencies, positive identity). The interview as a whole contained 41 questions related to the effectiveness of internal cohesion psychotherapy and 9 questions related to developmental assets.

### Procedure

Fifteen clients who were treated with ICP were contacted *via* telephone and were invited to participate in the current study. Ten of them accepted the invitation and took part in in-depth interviews voluntarily. The selected participants showed symptoms of depression and anxiety disorders. The data were collected *via* face-to-face semi-structured interviews, using two interviewing protocols. Before the interview, participants were informed about the scope of the project and their rights to participate. They were given a consent form to sign and their permission to record the interview was taken in advance. All interviews were conducted by two trained psychology practitioners and each interview lasted for around 1 h. The interviews were conducted in the Albanian language and took place in April 2022 in Prishtina. The ethical approval for the study was provided by the ethical committee of the multidisciplinary clinic “Empatia.”

### Data analysis

In this study we use thematic data analysis method, with a deductive semantic/manifest approach that involves exploring the data based on preconceived themes. Transcripts of audio recordings were used to analyze the explicit data. The data were coded by highlighting sections of the text such as phrases and sentences and categorizing them in codes. Seven codes or categories (psychotherapy experience, intrapersonal, interpersonal, professional and spiritual system, role of therapy, and developmental assets) resulted from the coding process and each category contained 3 to 12 subcodes (a total of 50 questions). Each transcript was coded separately, and all transcripts were used for the summary statistics. The data were analyzed using MAXQDA 2022 (VERBI Software, 2021) software.

## Results

All 10 participants interviewed for this study indicated that their therapy experience had yielded substantial benefits. The benefits of internal cohesion therapy were noted for a variety of reasons, but the most common ones were being understood, being free to express oneself, and gaining new perspectives on one’s difficulties/problems. The psychotherapist in ICP is viewed as a significant enabling factor for a person to open up about their own experience. DGHF says: *“One thing I loved from the first sessions was that I felt free.”* The experience in the internal cohesion therapy sessions is described by AGGS as emotionally liberating: *“I felt more relieved after the second session; it seemed as if I had removed weights from my back, and I noticed that therapy began to help me. I was quite open-minded and after the third session I noticed that this (therapy) is helping me.”*

Another benefit of this particular therapy is that participants gained new perspectives in dealing with their challenges and improve the way they comprehend the problem (i.e., anxiety) and, as a result, their reaction to problem-related circumstances. For example, DGSK says: *“(The therapy) allowed me to see things in a new light. (Before) I just looked at things in a “straight line,” did not look left or right, could not think of logical reasons why the anxiety was occurring to me or why I was having this particular sensation, and now I view things very differently.”* This new perspective for problems gained from the therapy participation, improved the relationship with self and own worldviews. AGMZH says: *“I used to have a hard time finding joy in simple things…and my goal for the year 2022 is to find joy in small things, and I’m approaching it (life) from a different angle … I’m taking pleasure in the minor pleasures of life…. I’m rediscovering a joy that I have not felt in years….”* The idea of “discovering self” is also mentioned by AGVF as a benefit of the therapy: “*I have learned a lot about myself… I am embracing and accepting myself as I am, beyond social constructs.”*

Participants reported largely positive changes in the intrapersonal system, which can be linked back to pre- and post-treatment, demonstrating that therapy played a significant influence in transforming participants’ intrapersonal communication. The participants demonstrated that they were able to have more positive self-esteem as a result of the insights they obtained in the internal cohesion therapy. One of the key reasons is that the therapy made them aware of the positive qualities they have. *“If I would rank it (the self-esteem) on a scale of one to ten, it was probably three or four before, and now it is eight-nine,”* AGAV indicates that therapy altered her self-esteem. The participant mentions exactly the objective voice coming from the psychotherapist as a reason for this improvement. *“I needed to hear from someone else, from a different standpoint, so I could see things from a different perspective, to see my situation from someone else’s perspective, not only from my own,” s*he adds. AGDZ emphasizes the role of therapy in self-esteem as well: *“In conversations with the psychotherapist he told me that I am an articulated person…and I said wow, you are right (to the psychologist). Sometimes you need to hear that from someone beyond your circle. This helped me because when you get on the black hole (negative thinking), you know that family members love you, even if you are the worst person in the world.”*

One of the ways this therapy helps clients improve their self-esteem is by assisting them in using their self-regulation skills to improve their approach to circumstances. AGGS links the change with self-regulation skills: “*I could have been the best at something (especially at university), but I could not learn. I began to worry about whether I would be able to graduate from university, what would happen if this (scenario) happened, and so on. I began to worry a lot about it, and now my eyes have been opened. And I believe there are more positive affirmations now than there were before when I used to give myself negative affirmations.”* AGGS gives high importance to external assets such as boundaries & expectations (especially adult role models) and constructive use of time. The participant believes that the model given to a child is crucial in the child’s development: *“Most of the reason why I have the self-esteem to achieve what I want is because of the example my mom gave to me.”* She adds that creative activities and youth programs are important because *“they have broadened the horizon of my perspectives.”* The use of self-regulation skills, in this case, lies in the idea that she actively and consciously substitutes negative thoughts with positive ones, with the idea of altering her situation. Similarly, AGDK points to this change and the potential use of self-regulation skills. She claims that it was the process of job searching that negatively influenced her self-esteem. She mentions that after the therapy, she does not allow this to influence her self-*esteem: “It does not mean you aren’t valuable if you aren’t valued in a job interview,” sh*e adds. It’s more of a deliberate blockade of those negative thoughts in her situation than a replacement of negative conceptions with positive ones (at least not mentioned here).

The improvement in self-esteem is noticeable. In some cases, not only the clients are aware of their change, but this is also reflected by other people around them. DGSG says: “*I am extremely happy, besides me, my mom too, yesterday when I told her that I will come for this interview, she said, I finally gained my daughter back. “*. Motivation, on the other hand, is the most difficult to influence among the three notions. Still, people attribute changes in their motivation levels to ICP: *“The fact that I am behind on my exams has left me with less time for hobbies, but I have a greater drive to do things*.” - adds AGGS. Furthermore, AGGS reports that the internal cohesion therapy has increased her motivation by 90%. “*Last year, especially, my mother tried everything, all methods…she tried everything, but nothing worked. “This is the first time that reality has altered (as a result of internal cohesion therapy) …”* - AGGS. DGSK also states that her motivation was previously quite low but has since greatly increased. *“I had no motivation for anything …* (now) *I have seatbacks, bad moments, but 95% of the time, I do things.”* -DGSK. When another participant, AGFH, was asked if treatment had helped her with her motivation levels, she responded, “*Very much*,” adding, “*It is still early, I need to develop further, but it had an effect.*” AGAV, also provides indications on the positive impact that the therapy had on motivation levels, as it says: “*It affected my motivation. Since I started the therapy, my motivation is much better, it was really low….”*

A similar reflection process that resulted in the person directing the purpose of its actions toward inner motivation is the case of AGMZH: “*I used to do things to make my father proud, now I am making things for my own, and it affected a lot (the therapy), no matter how bad I feel, I get up and carry on with the activities.”* Besides, AGDK and AGDZ indicate that the therapy has made them more resilient. “*I try not to fall into the laziness trap, and when I do, I make extra efforts to go to training on the days when I am the laziest…” - AGDK. AGDZ adds: “I used to get in my cave,”* but now *“I started to see the light. I’m more motivated.”*

Besides the intrapersonal system, one of the areas most affected by the client’s anxiety and/or depression are relationships with others, both family members and people beyond kin, i.e., friends. Clients’ experiences with ICP helped to improve those relationships or be reflexive about relationships and the efforts to pursue them or not. In this regard, AGGS describes her relationship with her mother as a “c*atastrophe*” before the experience in therapy. AGGS further says: “*When I got 18, it all started (the broken relationships). I accumulated a lot of hate, and even though I did not want to, I had too much negativity. I did not do it on purpose. I started to ruin healthy relationships with friends and family.”* AGGS considers that young people in Kosovo are influenced by society and links this to an internal asset such as Social Competencies, especially Resistance Skills: *“My circle of friends influenced me, and this negatively affected my life. No matter how smart you are it is hard to resist…you want to fit in and be accepted.”* When asked about relations with her mother in the present, she states that she still struggles with it, but it is “*much better*.” She gives credit to the ICP for why she started to get better in relationships with others, mostly because she said: *“I started to feel good. I do not have that much negativity on me…psychotherapy helped me to feel good, which helped me in my relationships with others.”*

AGFH says that for a long time she had an “*extended circle of people”* which she started losing as she says, “*for different reasons, people change, misunderstandings happen.”* Now she perceives her experience with ICP as useful as she says: *“Psychotherapy has opened my eyes because it showed me that not everything is as it looks*’. *I learned from the previous experiences; a lot of relationships were broken because I tried to make people behave according to a standard that I considered “good friendship.”* Moreover, AGFH considers that the effects of the therapy on her intrapersonal system helped her reflect and behave accordingly in relationships with others. “*Psychotherapy has helped me because it helped me to prioritize myself. I am thinking more positively about myself. It helps me that even other people perceive me as a nice and positive person, as I am*.”

AGAV, also indicates a positive role of the therapy as she says: “*Before starting with the psychotherapy, I was very isolated, I was self-isolated, as I always had issues to start a conversation, and I got very closed, now I am better, because I try to give love although sometimes, I find that difficult.”* Concerning this, she considers that being sincere and appropriately communicating with others is crucial which is linked to the honesty domain of positive values and the peaceful conflict resolution domain of social competencies: *“Although saying the truth is not easy, I prefer to say and hear it.”; “I think that in every relationship it is important to communicate peacefully and solve problems that way.”*

Another advantage of the therapy as DGSK reports is that she was able to talk freely about her anxiety with her family and friends. “*…I tell my mother, father, and friends that I am not feeling well today, that I am going to have an anxiety attack, and that they should not come to press me… This is something I’ve never done before.”* To AGDZ, the therapy was a good way to adjust her emotional reaction toward others, mostly because she perceives herself as the “*person who did too much for others,”* and now she says that she puts her needs first. *“…I’ve started to notice that I do not have to explain reasons for saying no when I do not want to go somewhere or hang out with someone*. AGMZ says that “*Now I can say sorry, I learned to talk about things, because when I get angry I do not like to talk, and I am noticing that I am way better.”* Because of this, she learned to let go of some relationships and some others to get better. One relationship she finds important and has improved is that with her brother. AGMZ says: “…*I am very connected with my brother, but our relationships fell apart. Thanks to psychotherapy and self-reflection, I managed to improve my relationship with him, which is one of the most important*.” Family support as an external asset is one of the most important development assets according to this participant: *“I believe every person needs support from family because it is fundamental for everyone.”*

Participants reported that psychotherapy has helped them in their professional aspect as well, through its influence on their approach to goals. When asked about three-time perspectives, they report changes that are attributed to psychotherapy. In this direction, AGGS states that in the past (it refers to the time before the therapy), objectives were built to fail purposely, while things have changed due to therapy. AGGS says: *“The goals were flimsy… I did not want to do anything, so I made up goals for myself, but once I started coming here (to therapy), I started setting reasonable goals for myself.”* In this line of thought, she mentions the relevance of internal assets such as the achievement motivation of commitment to learning factor: *“When I was motivated, I achieved what I aimed for. When I lacked motivation, I did not achieve anything.”*

Another participant, DGBS, shows that through therapy he managed to set more realistic objectives, by removing the self-pressure before a certain activity. When asked about what would be different now, DGBS said: “*I would not force it; instead, I’d attempt to be more direct in my communication and avoid putting too much pressure on myself about how things will turn out ….”* DGBS considers that communication is linked to social competencies as an internal asset. However, according to him, the latter is not important since they restrict the form of communication*: “social competencies seem to tell us how we should act, instead of how we want to.”* DGBS further reports that the approach toward goals changed positively: *“…the way I approach my goals is more positive.”* It is important to emphasize that the same participant states that internal PYD assets such as Positive Values are essential: “*the more positive values you have, the more positive your approach towards self and others.”*

AGFH demonstrates that she was able to alter her behavior toward responsibilities as a result of therapy. She used to panic when faced with a task, but now she is more optimistic about the outcomes. AGHF says: *“Previously, I put a lot of pressure on myself; I had goals, but my attitude was negative. That’s changed….”* Furthermore, AGDZ, like DGBS and AGFH, states that her approach to her goals has changed over time as she has learned to slow down and not put too much pressure on herself. “, she says. “*When I’m feeling low, I help myself…. I did not allow myself to fall into that hole when I had those very high ambitions, but now I do things more slowly, I do not have to rush, and we do not know how long we have (in this world), so I’m living in the now.”* AGGS, on the other hand, emphasizes the role of therapy in changing her perception toward herself in terms of the professional system. She claims that in therapy she managed to see her potential: *“It was one of the things that the psychologist focused on the most in sessions… (now) I am aware of how much I had (negatively) changed my perception of my potential and capacity.*

Furthermore, participants report that therapy has managed to always put another perspective on the table. DGSK mentions that one of the reasons her approach toward school and the future has changed is because the therapy managed to give her another perspective on things: *“It made me have a positive approach to everything*.” In a similar line of thought, AGFH conveys that therapy helped her see other perspectives: *“I used to concentrate on negative things in work, not necessarily related to work, now I am much more comfortable with my work, I think I am doing a good job.”* The ICP and methods used in it is powerful in the sense that clients were able to transfer their reflections to their everyday life. “Words *that the psychologist said to me remained in my head, and sometimes when I say I do not want to go to university, I then remember what he said. And that was a great motivation, it pushed me and affected me. Even when I think I cannot, I push myself to do that activity, because of this and this, I will do it.” – AGAV.*

In addition to that, AGDK mentions the therapy’s impact on her self-esteem and perspective on things as factors that positively influenced her approach toward the profession. ADGK says: “*It helped a lot (the therapy). At first, it increased my self-esteem. For a long time, I did not care for work, but I have seen that I was harming myself with it…the therapy helped me to see things a little differently, to see the reality, the context we are living in, not just how I want to see things*.” In this regard, AGDK perceives the internal PYD asset, positive identity, including its domains such as personal power, self-esteem, sense of purpose, and positive view of personal future, as relevant assets for youth development. The improvement that results from motivation and a desire to grow professionally from the therapeutic sessions can have a positive impact on how a person perceives themselves and their future. AGDZ exemplifies this when she says: *“I was in a very bad state, I did not want to work, I was very passive in general. Now I am feeling much better about myself, I have more will, good ideas, more creativity, and I like this change….”*

In ICP, the spiritual system is defined by both forms of existence, life, and death as well as their beliefs about them. The clients that participated in this study demonstrated that the experience in ICP can produce a positive change in the individual’s approach to both death and life and thoughts associated with such concepts, especially when experiencing anxiety. DGFH shows improvements in her perception of both aspects (death and life) and attributes this change to therapy: *“I see life with colors. I have started to see life with colors because I used to see only darkness. I used to judge myself, life, the way I used to live…now I am calmer*.” About death, DGFH says, “*I used to be afraid of death, I used to be very afraid. I used to sleep to escape reality…I used to be afraid of silence. Now I am not afraid of silence anymore*.”

Regarding the spiritual system, results indicate that therapy helped participants replace negative thoughts with more positive ones. In this direction, DGFH claims that the therapy assisted her in dealing with feelings about life and death by making her aware of her positive qualities. “*It helped (the therapy) by making me aware about my positive attributes. It stopped my negative thinking…those negative thoughts*.” Moreover, AGGS states that there have been improvements in her approach toward life and death when compared to the past and emphasizes that this change is a consequence of psychotherapy: *“I started not to love life, especially in the last 3–4 months. I thought…I was digging in my head to find a reason to live, just a reason, a little spark…but I did not manage to find it. Now I am starting to get back, I am seeing life as something that I still have interest in*.” AGGS had anxieties about not having enough time in this world to do everything she wants, and it is psychotherapy that made her see her relationship with time and death differently. She says: “*I used to feel like I was on the finish line when in reality I was on the start. Psychotherapy has helped me to understand that.” Concerning* death and thoughts about it, AGGS has seen death “as *a salvation”* in her bad moments, but once she started regaining routine (motivation) she started getting away from those thoughts. She says: “*It is a pleasure to make coffee in the morning, to go to university. When my routine has shifted, I started to understand that life is okay.”*

Not only AGGS, but other participants as well, reported that psychotherapy has positively impacted the way they see life and death. AGFH, for example, indicates that she now sees life with much more optimism. *“Life is now in bits and pieces for me; I’m trying to reflect on every moment and how happy I am.”* In the past, AGFH did not have this positive outlook and saw life as “*dark…*.” The therapy helped her to see life more positively because it helped her to “*stay in the present*.” AGAV reports that life was perceived as “pointless” before psychotherapy whereas now she uses different strategies to keep herself interested and present: “*I say to myself, find three good things that you did today, and I try, no matter how small it is, to say this thing made me feel good today*.” The significance of living in the present is emphasized by other participants as well: “*What helps me is that I live in the present (because of therapy), and do not have thoughts about what will happen tomorrow. We all think about that, but when I used to think about it, I used to overthink and could not get out of it. Now it is something else.”-* DGSK. AGDZ on the other side, portraits like as a *“gift”* and tries to *“make the best of it.”* She argues that before psychotherapy she has been unmotivated about life: *“It was different (life). I used to say, how can I live like this, why am I here….*” She further adds: “*It was the therapy that gave me calm.*” She has a similar shift in perceptions about death. She says: “*I used to have a phobia of death. Now I think of it as a natural process, and I hope I will leave something good for the world before I die.”*

Positive Youth Developmental assets are linked with reported progress in psychotherapy. Hereby, participants who report a positive experience in ICP mention at least one PYD asset as important. When asked about relevant PYD assets in the Kosovo context, all participants besides AGVF (9 out of 10) reported that Support is the most relevant factor, with a strong emphasis on family support. The least mentioned asset within the Support category is Parent Involvement in Schooling, whereas Caring Neighborhood was not perceived as relevant by any of the participants. Most participants, including AGGS, DGSK, AGVF, DGBS, DGSK, DGFH, AGFH, and AGAV (8 out of 10) report that the Boundaries & Expectations category within the external assets, with a special emphasis on Family Boundaries and School Boundaries, is a relevant factor. Whereas most of the participants including AGDK, DGFH, AGFH, AGGS, DGBS, DGSK and AGAV (7 out of 10) consider The Constructive Use of Time, especially Youth programs, as a relevant asset in the Kosovo context.

Regarding the internal assets, positive identity is rated as the most relevant by most participants, including AGDK, DGFH, AGGS, AGFH, DGBS, DGSK, and AGAV (7 out of 10). Most of these participants point out self-esteem as the most relevant internal asset and the second one is a positive value (6 out of 10 participants, including DGFH, AGAV, DGSK, AGGS, AGFH, and DGBS report on its relevance). Caring and integrity were also frequently used by participants, while DGFH, AGFH AGGS, DGSK, and AGAV state that commitment to learning and social competencies with an emphasis on resistance skills are relevant to the Kosovo context. On the other hand, two participants (AGFH and DGFH) stated that all of the factors and their domains are important for youth development.

Most participants declare that ICP has influenced several aspects of their lives. Moreover, participants emphasize the role of Positive Youth Development (PYD) assets in their psychotherapy process. AGAV mentions that therapy influenced a positive change in intrapersonal and interpersonal levels: *“Therapy has improved the way how I approach myself and others. It affected all aspects of my life.”* About this, the participant emphasizes that she is aware of the role of internal developmental assets such as interpersonal competence domain of Social Competencies factor: “*I believe that I highly empathize with others in every situation”* and integrity domain of Positive Values factor *“I stand up for my beliefs and behave accordingly.”* Regarding this, AGFH also reports that therapy helped improve her relationship with herself and others: “*I am working with myself with the help of the lessons I got in psychotherapy, and I am automatically reflecting my change to others… coworkers, family members, friends, as well as my cognitive processes (thoughts and self-reflections).”* Besides, she considers that family support and positive communication domains of Support factor are important assets, stating that *“family support and positive family communication impact the way I grew as a person…4–5 first years influence the whole future because based on this we build expectations, boundaries for myself and others, positive values, social competences, and especially positive identity.”*

Furthermore, DGSK considers that psychotherapy helped her reflect and understand herself better: *“I reflected a lot, from the simplest things to the more complicated ones. This helped me know myself, although I am still in the process.”* She adds that the focus is on herself: *“Now I pay attention to what affects me, and I do not accept what negatively influences me… and I am getting a lot of positive things from this reflection.”* Associating this statement to the internal PYD assets, DGSK highlights the relevance of resistance skills domain of Social Competencies factor, integrity domain of Positive Values factor, and personal power domain of Positive Identity factor: *“Resistance skills are present while I can resist towards others’ expectations… I also defend my beliefs, while I do not have a better example of how to perceive a situation and act towards it.”* On this matter, AGDZ states that therapy has a long-term effect *“it is not like medications that make you feel better in 5 min, its effect is longer…now I think more rationally, and I do not react quickly.”* She adds that her progress is reflected in others as well *“my dad says that now he can talk sincerely with me without the fear of any emotional reaction.”* AGDZ also states that she has always felt the support of her family *“my parents have always been there for me…my father always says that I can speak with him as with a friend.”* Hence, she finds the family support domain of the Support factor as the most important external PYD asset that mediates her progress.

Similarly, AGDK mentions the Support factor as a crucial asset that affects all other domains *“if there is support, everything else is easier to achieve.”* As regards psychotherapy, she reports that with the support of her family (husband and brother) she is aware of positive and negative feelings *“I used to have the fear of being judged,* e.g.*, in a room with family members where no one is feeling cold but me, I could not talk about it.”* Whereas now she says: *“It does not matter if no one is feeling like me, what I feel is important.”* On the other side, other participants report that psychotherapy is effective, but also necessary throughout life, especially when dealing with changes. For instance, DGBS says *“Psychotherapy is effective for me, but I am one of those people who constantly need to talk in a place where someone listens…I am in the process of knowing myself…that’s why I think the time I spent in psychotherapy is worth it.”*

In addition to that, DGBS considers that Constructive Use of Time is one of the most important assets *“I have the right to decide how I want to spend my time because if I spend my time efficiently, I’d feel better.”* Like DGBS, DGFH proclaims that she needs psychotherapy throughout life *“I need someone who listens and understands me without the need to comment about what I am saying.”* She adds: *“family and friends love you, but they are not engaged in the conversation… they often interrupt….”* According to her, the external PYD asset such as support is fundamental and it affects self-esteem as well: *“self-esteem is low as a result of the environment we grew up in, starting from the lack of support from family, friends, school, neighborhood, and society.”*

## Discussion

This qualitative study aimed to explore the effectiveness of Internal Cohesion Psychotherapy in treating young clients with depression and anxiety with a focus on the role of the developmental assets from the perspective of PYD. To address this research question, 10 participants were interviewed with a protocol designed to cover both the effectiveness of the ICT (see Uka, 2022 for a comprehensive overview) and the presence of developmental assets in clients’ life. The findings confirmed the effectiveness of ICT in treating clients with depression and anxiety and identified additional positive outcomes that are directly linked to psychological well-being. Specifically, participants were satisfied with the improvement they experienced in the intrapersonal and interpersonal systems after receiving ICP treatment. When comparing the past with the present time, they report positive changes, mainly indicating better communication with themselves and with important figures in their lives. With the help of ICP, participants dealt with their events in the past, which led to acceptance (a central construct in ICP perspective), built better relationships in the present with each system, and created more constructive and achievable goals for the future. As such, it can be concluded that most of the clients reached internal cohesion, thus improved their psychological well-being according to the ICP perspective established by Uka (2022).

In addition to reporting significant positive experiences with Internal Cohesion Psychotherapy, our findings further contribute to and support existing evidence about the importance of developmental assets in optimal emotional and mental well-being (i.e., Adams et al., 2019; Uka et al., 2021; Wiium et al., 2021). In line with the goals of ICP theory and practices (Uka, 2022), significant positive changes were reported in clients’ intra- and interpersonal systems, which in turn showed positive effects on other systems as well (professional and spiritual ones). More specifically, clients highlighted observable positive changes in their self-esteem, self-regulation skills, and motivation, adding that these changes were reflected firmly in their relationships with family members, peers, and colleagues in their workplace. Similar to these findings, other studies have shown positive significant relations between perceived and exemplified levels of motivation and resilience (Benson et al., 2011; Manrique-Millones et al., 2021; Uka et al., 2021). On the other hand, improved interpersonal relations were the main highlighted outcome from the participants’ point of view, while better intra- and interpersonal relationships and emotional regulation, as well as improved communication approaches, were linked to social competencies as internal and family as external Positive Youth Development Assets (Benson et al., 2011; Ismail et al., 2015; Manrique-Millones et al., 2021). As such, the positive effects of ICP treatment can go beyond the factors evaluated in this study and influence the overall mental health and well-being.

Findings from the current study offer thoroughly elaborated positive changes in the clients’ professional systems after completing their ICP process. While commenting on the positive results achieved in the professional system, clients once again emphasize the importance of various internal assets such as achievement motivation, social competencies, positive values, and positive identity (Benson et al., 2011; Manrique-Millones et al., 2021). Responsive to Internal Cohesion Psychotherapy (Uka, 2022), clients reported a transformative shift in regard to the way they approach their expectations and goals for the future and the role of internal assets in achieving such goals was often acknowledged. With motivation acting as a mediator during sessions, clients frequently reflected on their newfound tendencies to set real and achievable expectations for the future. Additionally, improved self-esteem and self-regulation as clients started to get in touch with their potential again, helped them reach their professional goals.

Lastly, the spiritual system, a system which is described as “finding meaning in life” (Uka, 2022) was reported to have been positively advanced on account of the therapeutic process. Although there are fewer concrete results noted by clients regarding the spiritual system, they admit that the ICP produced an important shift on the conceptualization of life and death, which resulted in reduced fear/anxiety from unpredicted events or threats which lead to death. However, a better-established spiritual system was also mentioned along with other developmental assets, such as the constructive use of time, while the religious community aspect of the asset was considered important for some of the clients. In line with accumulated evidence (Tepper et al., 2001; Bosworth et al., 2003), ICP argues that engagement in religious and spiritual activities helps individuals deal with adverse experiences in life.

In compliance with the Dynamic Skills Theory (Fischer and Bidell, 2006) as an integrative approach that describes mechanisms of development considering a variety of interdependent factors, our findings indicate that systems of ICP (Uka, 2022) are related to developmental assets and cannot be treated as independent from resources that an individual has. Both internal and external assets are shown to play an important role during the psychotherapeutic process. Therefore, values, relationships, resources, and skills are the assets that ease and contribute to the higher effectiveness of Internal Cohesion Psychotherapy. As expected, the intervention in a certain asset or system showed a positive result on other assets or systems as well, confirming the principles of dynamic skills theories (i.e., Fischer and Bidell, 2006). Thus, our results are promising in terms of incorporating developmental assets to promote mental health and psychological well-being among young adults as well as to maximize the effectiveness of ICP.

### Limitations, implications, and conclusion

To our knowledge, this is the first paper that links the effectiveness of a psychotherapy approach with developmental assets by using clinical samples. As such, when interpreting the findings, some limitations should be noted. First, this study has a relatively small number of participants, and the sample has not been matched in terms of gender. This is due to the fact that ICP is a new approach and the selection criteria included only participants who took more than five sessions and completed their therapeutic sessions. Second, this study only focuses on an integrative psychotherapy approach, such as ICP, and results cannot be used to generalize the effects of developmental assets on the effectiveness of other psychotherapies (which do not use a system-based approach). Third, the design of the study is qualitative, thus limiting the interpretation of causes and effects. Thus, future studies can address such limitations, by implementing longitudinal mixed study designs (i.e., different time intervals, preferably before, during, and after the therapy), which include larger samples with even gender distribution and assess other psychotherapy approaches as well.

Beyond limitations, the current study has some important theoretical, practical, and research implications. In terms of theoretical implication, findings confirm the interrelation between psychological factors and systems, providing evidence for the dynamic skills theories and approaches. Thus, intervention programs can also benefit from the findings that support ICP claims for factors and systems not being independent. For example, intervention programs that aim to improve academic performance among youth can benefit from these findings and involve in their intervention intrapersonal and interpersonal systems or developmental assets. This implies that intervention in specific factors and systems yields positive results alongside other factors and systems as well. The results of the current study reflect the impact of ICP approach in clinical practice, in treating clients with depression and anxiety disorders, thus expanding treatment alternatives in clinical settings. This is preliminary evidence which speaks in favor of eclectic approaches in treatment of anxiety and depression, which are more preferred among clinicians (Boswell et al., 2010; Zarbo et al., 2016). Based on our findings, we can conclude that addressing experiences and events from the past and present as well as discussing openly future expectation has a positive impact on mental health of clients.

To conclude, this study also opens a new path of research on the use of developmental assets to enhance the effectiveness of psychotherapies and can be a foundation ground for different intervention programs, which aim to promote mental health and well-being among youth.

## Data availability statement

The raw data supporting the conclusions of this article will be made available by the authors, without undue reservation.

## Ethics statement

The studies involving human participants were reviewed and approved by the ethical committee of the multidisciplinary clinic “Empatia.” The patients/participants provided their written informed consent to participate in this study.

## Author contributions

FU conceptualized the study and was responsible for the final draft of the manuscript. SG prepared ICP interviewing protocol and data analysis plan. AG conducted the interviews, performed data analysis, and wrote the methods section of the manuscript drafts. DG conducted and transcripted interviews and performed data analysis. AM wrote the discussion section of the manuscript. AK, AS, VS, and IS transcripted the interviews. VP was involved in the initial draft of the discussion section of the manuscript. NW provided critical revisions of manuscript drafts. All authors contributed to the article and approved the submitted version.

## Conflict of interest

The authors declare that the research was conducted in the absence of any commercial or financial relationships that could be construed as a potential conflict of interest.

## Publisher’s note

All claims expressed in this article are solely those of the authors and do not necessarily represent those of their affiliated organizations, or those of the publisher, the editors and the reviewers. Any product that may be evaluated in this article, or claim that may be made by its manufacturer, is not guaranteed or endorsed by the publisher.
